# Hematological Malignancies in Older Patients: Focus on the Potential Role of a Geriatric Assessment Management

**DOI:** 10.3390/diagnostics14131390

**Published:** 2024-06-29

**Authors:** Santino Caserta, Gabriella Cancemi, Silverio Loreta, Alessandro Allegra, Fabio Stagno

**Affiliations:** Division of Hematology, Department of Human Pathology in Adulthood and Childhood “Gaetano Barresi”, University of Messina, via Consolare Valeria, 98125 Messina, Italy; santino.caserta@polime.it (S.C.); gabriella.cancemi@polime.it (G.C.); lrtsvr01d11f158p@studenti.unime.it (S.L.); alessandro.allegra@unime.it (A.A.)

**Keywords:** geriatric assessment management, GAM, hematological malignancies, lymphoma, leukemia, chronic myeloid leukemia, multiple myeloma, elderly, aging, cancer, clinical trial

## Abstract

Geriatric assessment management is a multidimensional tool used to evaluate prognosis for clinical outcomes and targets for interventions in older adults with cancer receiving chemotherapy. In this review, we evaluated the possible application of geriatric assessment management (GAM) in hematological malignancies. In older patients with Diffuse Large B Cell Lymphoma, GAM might be helpful in both predicting planned hospital admissions and improving quality of life. In chronic myeloid leukemia, the Charlson Comorbidity Index demonstrates how comorbidities could affect treatment compliance and overall outcomes. In multiple myeloma, the application of different scores such as the International Myeloma Working Group Frailty Index and the Revised Myeloma Comorbidity Index can identify frail patients who need suitable interventions in treatment plan (reducing drug dose or changing treatment). Therefore, including GAM in the management plan of older patients with hematological malignancies may direct and optimize cancer care.

## 1. Introduction

Cancer is considered an age-related disease, with 60% of diagnoses made in patients 65 years of age and older [1]. Moreover, aging is considered also a major risk factor for the development and progression of cancer, together with related conditions such as obesity, smoking, and alcohol consumption [2]. Older people usually have a worst performance status and exhibit a worse prognosis for several cancers. Evidence has been reported that some mechanisms underlying cancer and aging are comparable: these pathways include altered metabolism, telomere shortening, proteostasis loss, decreased nutrient sensing, and epigenetic modifications [3,4]. Given the tight relationship between aging and cancer, epigenetic variables affect the entire course and development of both conditions [5]. Among hematological malignancies, most patients with non-Hodgkin lymphoma, leukemia, and multiple myeloma are diagnosed late in life with a median age of 67, 67, and 69 years old, respectively [5,6]. Elderly patients with blood cancer frequently have age-related vulnerabilities that make their care more difficult. In fact, frailty, cognitive impairment, and functional reliance are common, and are linked to worse outcomes because of increased treatment toxicity, unforeseen hospitalizations, and higher death rates [6].

### 1.1. Geriatric Assessment Management in Cancer

Cancer organizations such as the American Society of Clinical Oncology (ASCO) recommend geriatric assessment management (GAM), a multidimensional assessment to evaluate patient risks, prognosis for outcomes, and targets for interventions, in older adults with cancer receiving chemotherapy [7]. Evidence from observational studies suggests that GAM accurately identifies age-related vulnerabilities, directs their care, and influences treatment choices. Moreover, GAM might lower mortality, hospitalizations, and functional decline in the older adult population, also predicting outcomes for older cancer patients [8,9]. Recent studies suggest that GAM-guided therapies could enhance both quality of life and patient–caregiver communication [10]. According to the ASCO guidelines, GAM should be a part of the management plan for all cancer patients above 65 years of age, to address deficiencies with suitable interventions and utilizing outcomes to guide decisions about cancer treatment [11,12]. GAM also improves communication, discussions about aging-related concerns, and patient/caregiver satisfaction with care. In terms of cancer care optimization, GAM might be helpful in achieving two primary endpoints: i. to obtain a discernible increase in the number of multidisciplinary interventions and ii. to encourage a shift on how clinicians approach management decisions (such as reducing dose in chemotherapy schedules, encouraging a better adherence to therapy, and defining better care goals). In brief, GAM can influence the decision-making process, helping to select the best therapy and providing detailed information about the goals of care. As a result, this management results in clinical care that avoids treating fit patients too little or in over-treating weak patients [13,14]. While the precise processes on how GAM functions in various situations and contexts are still being studied, it is widely accepted that GAM should consider factors such as diet, social support, comorbidities, mental and physical well-being, and polypharmacy [15]. The Chemotherapy Risk Assessment Scale for High-Age Patients (CRASH) score is one of the tools used to stratify older patients with cancer into four risk categories: low, medium-low, medium-high, and high. Evidence has showed that, even in the lowest category, there is still a considerable pooled risk of severe toxicity and points out that older patients receiving chemotherapy should be closely monitored [16]. The introduction of the CRASH score in both academic and communal context allowed researchers to compare them [17,18,19,20,21]. To this address, the CRASH score can be a useful stratification tool to better determine which toxicities based on Common Toxicity Criteria for Adverse Events (CTCAE) might call for treatment modifications [22].

#### 1.1.1. Function and Mobility

Older cancer patients’ function and mobility can be assessed by objective measurements or self-reported data. The capacity to perform instrumental activities of daily living (iADLs) and activities of daily living (ADLs), as well as the number of falls of a person in the previous six months, are self-reported measures [23,24]. ADLs represent the fundamental self-care tasks needed to preserve independence at home (such taking a shower) while iADLs include the more sophisticated tasks required to preserve independence in the community (like going shopping). Higher treatment tolerance and lower survival rates have been linked to older cancer patients’ demand for help with instrumental activities of daily living (iADLs). Objective tests like the Short Physical Performance Battery (SPPB), the Timed Up and Go (TUG) test and the Timed 10-Meter Walk Test are frequently utilized to evaluate function and mobility in older people. Guidelines suggest performing the evaluation of ADL, iADL, and at least one additional objective measure of function and mobility when evaluating an older adult with cancer prior to treatment. The SPPB is a test that measures gait speed, balance, and strength in older persons, allowing evaluation of lower extremity function and mobility. It is also used to forecast mortality, hospitalization, admission to home nursing, frailty, mobility impairment, and ADL difficulty. A low SPPB score (<10 at baseline) is highly predictive of mobility handicap in older persons and is linked to statistically significant disabilities. Guidelines recommend potential interventions in cases of limitations in function and mobility, including home safety evaluation, promotion of physical activity and exercise, referral to physical medicine and rehabilitation, occupational therapy, and a geriatric-trained clinician or primary care physician [25].

#### 1.1.2. Comorbidities

Comorbidities are frequent in older people and can affect treatment adherence and tolerance, life expectancy, and cancer prognosis. Guidelines suggest the use of scales such as the Charlson Comorbidity Index (CCI), the Cumulative Illness Rating Scale (CIRS), or the Hematopoietic Cell Transplantation-specific Comorbidity Index (HCT-CI), to assess the risk of mortality associated with comorbidities. CCI aims to predict risk of death within 1 year of hospitalization for patients with comorbidities [24]. It has also been demonstrated that the CCI produces concordant predictions or has contemporaneous validity with several other prognostic scales. Moreover, it seems to be a reliable indicator of long-term mortality in a variety of clinical populations, such as older patients with cancer, and patients undergoing surgery, intensive care unit (ICU), and trauma. CCI seems beneficial in distinguishing significant differences among patients with the same medical diagnosis, in addition to offering a valid assessment of the patient’s particular clinical state [25].

#### 1.1.3. Social Functioning and Support

The physical and emotional well-being of cancer patients has been linked also to the availability of social support. Social support networks are essential for older persons with cancer to maximize treatment success. It has been found that one of the most important predictors of older individuals’ death is a lack of social connections. Therefore, before beginning anticancer therapy, healthcare providers should perform a thorough assessment of the social support of an older adult, considering a patient’s living situation, financial situation, and the existence and suitability of a caretaker. In detail, the self-administered Medical Outcomes Study (MOS) social support survey employs four subscales (emotional/informational, tangible/instrumental, pleasant social interaction, and affection) and one overall index to measure the availability of assistance in multiple areas. It is also important to verify if the patient is a caregiver for other people and if receiving cancer treatment would affect his ability to do so. Guidelines recommend different potential interventions in cases of inadequate social support, such as referral to social work for a comprehensive evaluation, review of home safety and issuance of medical alert devices, consultation with psychiatry or psychology, screening for elder abuse and caregiver burden, and spiritual care [25].

#### 1.1.4. Cognition

Older cancer patients with a cognitive impairment are more likely to experience sadness, functional reliance, and a higher death rate comparable to that of those who receive a cancer diagnosis. Throughout their therapy, individuals with cognitive impairments should be well-supported by a skilled multidisciplinary geriatric oncology team [26,27,28]. Furthermore, in the context of cognitive impairment, the capacity to balance the advantages and disadvantages of anticancer therapy and to follow treatment guidelines needs to be carefully evaluated. Guidelines suggest utilizing fast assessments and straightforward techniques to test any cognitive impairment prior to starting treatment with the Mini-Mental State Examination (MMSE) and the Montreal Cognitive Assessment (MoCA). The MMSE is a screening test consisting of 11 items that is used to measure objectively cognitive impairment severity and track changes in cognitive function over time. The quick screening test called MoCA can identify moderate cognitive impairment with high sensitivity and specificity [29,30,31]. Similarly, older persons can be screened for cognitive decline using the five-point Mini-Cog, which consists of a clock drawing test and a three-word recall exam. Lastly, a six-item weighted survey called the Blessed Orientation Memory Concentration (BOMC) test assesses patients’ orientation, registration, and attention to identify dementia. Another instrument might be machine learning (ML), since studies have demonstrated that artificial intelligence can be applied to elderly patients with both cancer and cognitive impairment to predict hospital admissions, health care planning, and chemotherapy management [32,33,34,35,36]. Fatigue, sadness, anxiety, underlying brain tumors, endocrine abnormalities, dietary deficiencies, alcohol use, and sleep disorders can also complicate the assessment of cognitive performance. Thus, additional assessments such as brain imaging, neuropsychological testing, and examination for vitamin B12 deficiency and thyroid dysfunction may be necessary if a dementia is suspected. In addition, chemotherapy may also contribute to cognitive deterioration in cancer patients and cognitive impairment might last for several months or years after treatment, depending on the underlying cause. Therefore, other potential interventions for patients with impaired cognitive function include involving the patient’s family or caregiver, minimizing any inappropriate medications, implementing delirium prevention measures, assessing the patient’s ability to consent to treatment, and identifying a healthcare proxy [25].

#### 1.1.5. Psychological Problems

The prevalence of anxiety and depression can significantly affect patient’s capacity to undergo life-sustaining cancer therapy. While poorer physical function and loss of independence represent the main risk factors contributing to distress, impaired mobility and functional status, inadequate social support, cognitive impairment, polypharmacy, multimorbidity, and cancer-related pain are independently associated with clinical depression [28,37]. The Geriatric Depression Scale (GDS) is a viable tool when screening for depression elderly adults without or with mild-to-moderate cognitive impairment. Since depression and cancer-related fatigue often coexist, those patients who complain of exhaustion should be evaluated. The NCCN Distress Management Panel created the well-known NCCN Distress Thermometer (DT) which together with the Hospital Anxiety and Depression Scale is useful to determine if cancer patients experience issues in five domains: family, practical, emotional, spiritual/religious, and physical [38,39,40,41,42,43]. Potential therapies for those with depression or psychological discomfort include referrals to integrative medicine, counselling, psychiatry or psychology, addition of anxiolytics or antidepressants, support groups, and spiritual care [25].

#### 1.1.6. Nutrition

Malnutrition, or nutritional deficiencies, are dangerous conditions frequently underdiagnosed in older persons in general and especially in cancer patients. In hospitalized cancer patients, inadequate nutritional status is linked to a higher risk of severe hematologic toxicity, a higher risk of mortality, poor chemotherapy tolerance, and a longer length of hospitalization [44,45]. Cutoffs such a body mass index <22 kg/m^2^ and the percentage of unintentional weight loss in the preceding six months are used by the malnutrition universal screening tool and make it simple to identify those individuals at risk for treatment intolerance and malnourishment. A validated technique is the Mini-Nutritional Assessment (MNA) that is capable of showing which groups are at risk of malnutrition, have enough nutrition, or are malnourished in terms of protein and calories. Additionally, it was discovered that the MNA may predict hospital costs and death [46]. Guidelines recommend a nutrition consult, target dietary interventions, oral hygiene, additional nutrition, occupational therapy for assistive devices, speech therapy, swallowing evaluation, oral/dental evaluation for dentures, screening for food insecurity, social/caregiver support, and assessment for appetite stimulants/nausea control/calories/protein [25].

#### 1.1.7. Polypharmacy

Polypharmacy refers to using multiple medications (>5) [47]. While polypharmacy can be an issue in any age group, in older people it represents a greater risk because of comorbidities. In fact, comorbidities treated with more than one medicine may raise the risk of nonadherence, drug–drug interactions, and adverse drug responses, resulting in functional decline and geriatric syndromes [48]. Adverse drug interactions can also be caused by changes in the pharmacokinetics and pharmacodynamics of drugs in the elderly population. An increased risk of falls might be due to the use of potentially unsuitable drugs, particularly hypnotics, sedatives, antidepressants, long-acting benzodiazepines, and other inappropriate psychotropics, as well as medications with anticholinergic effects. Guidelines suggest screening for polypharmacy with proven techniques, particularly in older persons undergoing chemotherapy regimens, since many drugs interact with other routinely used medications in this subset population [49]. Two widespread methods for assessing possibly inappropriate medication use in elderly people are the Beers Criteria and the Medication Appropriateness Index (MAI). Drug interactions, medication duplication, and medication underuse may be also assessed by using the Screening Tool of Older Persons’ Potentially Inappropriate Prescriptions (STOPP) and the Screening Tool to Alert Doctors to Right Treatment (START) criteria [50,51,52,53]. Based on the possibility of drug–disease interactions and the risk of toxicity in elderly cancer patients, the Beers Criteria identify unsuitable drugs with risks that may exceed benefits. The American Geriatrics Society has modified it with the goal of enhancing patient outcomes. The updated criteria categorize medications used in older adults into the following groups: (I) those that should be avoided in most older patients; (II) those that should be avoided in older patients with specific conditions; (III) those that should be administered cautiously because the benefits outweigh the risks; (IV) those whose dosages should be adjusted in accordance with renal function. Furthermore, while the START criteria include 22 evidence-based indicators to identify prescribing omissions in older people, the STOPP criteria are made up of 65 indicators for potentially inappropriate prescriptions, including therapeutic duplication, drug–drug and drug–disease interactions, and drugs that cause a major risk of geriatric syndromes. Prior to starting or altering a treatment plan, a medication review of all currently prescribed and over-the-counter medications may be necessary. When using some medications or classes of pharmaceuticals that are not advised for older persons, clinicians should carefully evaluate the indication for treatment, length of therapy, and dose (Table 1) [25]. Unfortunately, there are few studies on geriatric patients explicitly: for example, there are very few studies about the interactions of biological treatment for allergies with other geriatric medications [54]. Moreover, there were articles where the full text was not available for free, so it is reasonable that there is more evidence of the usefulness of GAM in older patients with hematological neoplasms than it may appear.

## 2. Discussion

### 2.1. Geriatric Assessment Management in Hematological Malignancies

**Figure 1:**
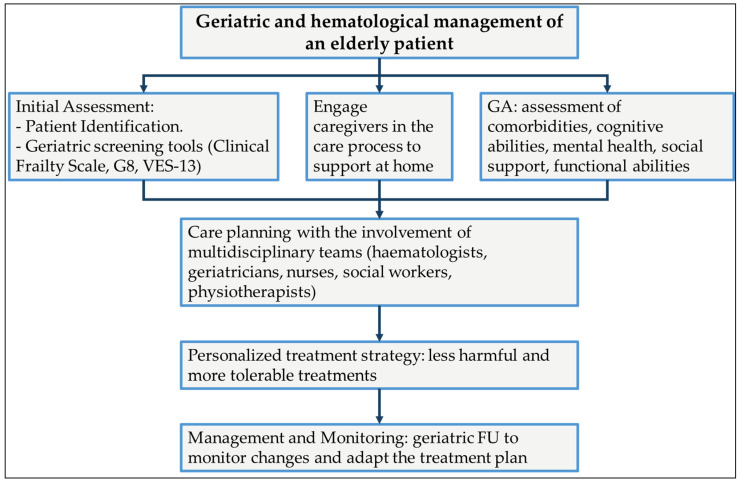
Flowchart for a systematic approach to the management of elderly patients with hematological malignancies, emphasizing the importance of geriatric assessments and coordinated care to improve treatment outcomes and quality of life. FU, follow-up; G8, Geriatric 8; GA, Geriatric Assessment; VES-13, Vulnerable Elders Survey-13.

The management scenario of hematologic malignancies has changed over recent years with significant advancements leading to less harmful and more easily endured therapies. Nevertheless, a personalized cancer treatment that meets the unique requirement of older patients remains a challenging task. In this view, a detailed assessment encompassing the aspects related to aging, comorbidities, cognitive and functional abilities, mental health, social support, and physical endurance is mandatory for selecting the most appropriate therapy for everyone (Figure 1) [55].

The care of frail and older adults is often complex and an approach that entails the assessment of elderly patients by geriatricians offers the advantage of enabling coordinated care with hematologic oncologists within the same clinical setting [56,57,58,59]. Limited investigations on GAM-guided interventions in patients with blood cancers have been reported. However, it has been demonstrated recently that GAM-based interventions carried out by a multidisciplinary geriatric team among patients (average age of 67) undergoing hematopoietic stem cell transplantation for blood cancers led to an improvement in one-year overall survival [56,60,61,62,63]. In a randomized study involving patients with hematologic malignancies (aged 75 years or older) deemed ineligible for transplantation and who received either geriatric consultation or standard care, no significant impact was observed on one-year overall survival. This outcome may be attributed to the fact that not all patients completed the geriatric consultation, due to organizational difficulties and scheduling issues, and just over half received follow-up visits. Key differences include the timing of the initial geriatric visit, which ensures a timely GAM-driven intervention, the number of visits performed and the follow-up period. However, no correlation was found between the number of visits and mortality rates [6]. Various geriatric screening tools, such as the Clinical Frailty Scale and the Frailty Index, have been developed and validated to detect and categorize frailty and to select patients who might benefit from a thorough geriatric assessment [57,58,59,60]. Several care models have been suggested for establishing a geriatric hematology clinic: for instance, in a geriatric oncology unit model, hematologists gain essential geriatric knowledge to conduct geriatric assessments, including the use of screening tools and evaluations for geriatric screening, possibly complemented by the involvement of adequately trained nurses. A second model involves a geriatric consultation or a co-management team that incorporates geriatric care into the patient’s clinical team through collaborations between a hematologist and a geriatrician with experience in caring elderly patients with hematologic malignancies. This team could be further enriched by including nutritionists, social workers, and physical therapists. A third model foresees the hematologist looking for geriatric consultations among healthcare personnel located within the same medical center or healthcare system, or from an external center. The successful implementation of these interventions in low- and middle-income countries (LMICs) necessitates not only an increase in staff but also an enhancement of the geriatric competencies of the entire hematology workforce (Table 2) [61].

#### 2.1.1. Focus on Lymphoma

Age-related functional decline and comorbidities may vary greatly in patients with lymphomas and may impact treatment outcomes. Further, they might go also unrecognized in clinical practice. Comprehensive geriatric assessments (CGA) are not usually employed in patients with lymphomas due to their time-consuming nature and the lack of validation as a standard treatment guideline. Therefore, it might be helpful to develop an appropriate risk stratification model for the elderly population [62,63,64]. The Italian lymphoma group categorized elderly patients with diffuse large B-cell lymphoma (DLBCL) into three groups (fit, unfit, and frail) using three streamlined geriatric assessment instruments: Activities of Daily Living (ADL), Instrumental Activities of Daily Living (IADL), and the Cumulative Illness Rating Scale for Geriatrics (CIRS-G), which were linked to patient age and validated within an external cohort [65,66,67,68,69]. Several retrospective studies explored the effects of the R-CHOP regimen, consisting of rituximab, cyclophosphamide, doxorubicin, vincristine, and prednisone, on elderly DLBCL patients. However, most of these studies found no significant correlation between dose intensity and survival in the older cohort of patients. One study suggested that doxorubicin dose intensities ≥85% correlated to poorer outcomes than intensities below 85% [70]. This phenomenon can be attributed to the increased treatment-related toxicities and early mortality rates among older patients exposed at higher dose intensities. Among studies on lymphomas, the GERIAD prospective study was designed to create a risk model using a simplified geriatric assessment along with other geriatric variables to predict event-free survival (EFS) in older DLBCL patients, and to evaluate how geriatric-defined non-fitness affects relative doxorubicin dose intensity (RDDI), treatment-related toxicities, and survival outcomes [69].

The results of the GERARD study showed that a geriatric risk model including age, functional condition, and comorbidities can effectively predict outcomes [71]. Elderly patients identified as non-fit faced increased risks of disease progression, mortality, symptomatic non-hematologic adverse events, and early cessation of treatment, irrespective of their age-adjusted international prognostic index (aaIPI) scores. Of interest, higher RDDI correlated with worse outcomes in non-fit elderly patients.

This latter study also suggested that patients who were considered fit had tolerable levels of non-hematologic toxicities and premature treatment discontinuations, making them suitable candidates for standard dose-intensity chemoimmunotherapy. Conversely, the major reason for early treatment discontinuation among intermediate-fit or frail patients was due to treatment-related toxicities such as febrile neutropenia, fatigue, and lung infections. Patients in the intermediate-fit or frail categories who received a reduced RDDI showed better survival outcomes than those with a higher RDDI, implying that lowering treatment-related toxic effects through a dose-reduced chemoimmunotherapy strategy could be effective to enhance short-term outcomes in elderly non-fit patients. 

All these data suggest that geriatric fitness may be a significant predictor of long-term outcomes in DLBCL, highlighting the need to develop new therapeutic approaches for non-fit patients. Further innovative treatment strategies involving new agents like bispecific antibodies are under active investigation for elderly or unfit patients with DLBCL, with early clinical results showing encouraging outcomes. Future randomized trials based on these novel strategies could provide optimal treatment options for this highly vulnerable subgroup. A geriatric risk model may prove useful in selecting patients who are most likely to benefit from these cutting-edge approaches [72]. In the phase III open-label, multicenter Integrated Geriatric Assessment and Treatment Effectiveness (INTEGERATE) trial, 154 elderly patients aged 70 or older with either solid tumors or DLBCL were split into two groups: 76 received integrated oncogeriatric care and 78 received standard care. The integrated care involved a GAM, which covered medication review, assessments of physical, comorbidity analysis, cognitive, psychological, and social functions, as well as evaluations of falls, nutrition, frailty, sensory impairments, advanced care planning, chemotherapy toxicity risks, and immunization status, followed by consultations and implementations based on the GAM findings. The primary endpoint of the trial aimed to evaluate any changes in health-related quality of life (HRQOL) over 24 weeks, using the Elderly Functional Index (ELFI) at baseline and weeks 12, 18, and 24. Secondary outcomes focused on functionality, mood, nutritional status, treatment adjustments, healthcare usage, and OS. Results suggested that integrated oncogeriatric care significantly improved HRQOL and reduced the incidence of unplanned hospital admissions as compared to standard care, though no differences in OS were observed between the groups. The GAP701 trial investigated whether GAM could be helpful in lessening severe (grade 3–5) toxic effects associated with chemotherapy. The trial recruited 718 elderly patients, aged 70 or older, who had incurable solid tumors or lymphoma and at least one non-polypharmacy vulnerability and who were initiating a new high-risk treatment. Patients were randomized to either a standard care group (*n* = 369) or an intervention group (*n* = 349). The primary endpoint was the incidence of any grade 3–5 toxic effects within three months of starting the new treatment regimen, with secondary outcomes focusing on the occurrence of falls and the management of polypharmacy. Data analysis revealed that fewer patients in the intervention group experienced high-grade toxic effects compared to those in the standard care group [73,74]. 

#### 2.1.2. Focus on Chronic Myeloid Leukemia

The significance of age as a prognostic factor in patients with chronic myeloid leukemia (CML) has changed in the era tyrosine kinase inhibitors (TKIs) [75,76]. In a clinical trial conducted by Breccia and colleagues, the effects of comorbidities were studied in 181 elderly patients over 75 years with CML, who were treated with imatinib (IM). This study utilized the CCI to determine how concurrent diseases impacted patient compliance and overall outcomes, as well as how the initial age of patients influenced clinical decisions. It was noted that the standard dose of IM was lowered at the initiation of treatment for 68 elderly patients, regardless of the initial assessment of comorbidities. This choice was based on the physician’s judgment solely: 43.6% of patients with a CCI score of 0 began treatment with a reduced dose and 33.6% of patients with a CCI score ≥ 1 also started on a reduced dose. When comparing the CCI stratification to the physician’s perception of the patient’s capacity to tolerate the medication (regardless of comorbidity evaluation), it was found that patients without comorbidities who started on a reduced dose showed a reduced rate of molecular responses. The study also revealed that comorbidities affected event-free survival and influenced median overall survival, although they did not affect the efficacy of the treatment [75,76]. Moreover, the study showed that treatment compliance was lower in patients who were initially administered a reduced dose: Patients who were originally treated with a conventional dose did not show any discernible changes in medication decrease. Independent of the first dose, discontinuations owing to toxicity happened in 44% of patients with a score of 0 and in 60% of patients with a score of ≥1, while patients who started at a lower dose had a higher rate of discontinuations due to toxicity than patients who received the full dose [76].

In another patient cohort, it was demonstrated how comorbidities could aid in treatment decisions among elderly patients treated with dasatinib (DAS). Initial application of the CCI or the Adult Comorbidity Evaluation-27 (ACE-27) identified patients who were at a heightened risk of experiencing pleural effusions and having poor drug compliance. The findings suggest that it would be reasonable to define an elderly patient using reproducible measures of frailty rather than solely based on the physician’s perception, thereby improving initial decision making in this patient subgroup [77]. 

A recent evidence-based study compared HRQOL among newly diagnosed CML patients treated with either IM or DAS. Results revealed only minimal differences in HRQOL between older patients treated with DAS or IM. Thus, elderly patients with CML appear to exhibit similar HRQOL profiles when treated with these two TKIs. Even if treatment-free remission (TFR) might not be the primary goal for this subset of elderly patients with CML, the use of second-generation TKIs could potentially expand the number of patients eligible to discontinue TKI treatment. As a result, initiating treatment with DAS may prove advantageous for certain older patients with CML who aspire to achieve a DMR [78]. In elderly patients, it is critical to tailor the most suitable TKI-treatment to ensure ongoing safety and adherence over the long term. In fact, adverse effects and coexisting conditions can be effectively managed by adjusting the medication dosage, which proves to be just as effective [79,80,81].

#### 2.1.3. Focus on Multiple Myeloma

Identifying and providing an appropriate treatment for frail elderly patients with multiple myeloma (MM) continues to be an unmet medical need. Older adults vary widely in their physical function, prompting the development of several frailty assessment tools specific to MM, including the International Myeloma Working Group (IMWG) Frailty Index, the Simplified Frailty Index, and the Revised Myeloma Comorbidity Index [76,82,83,84]. Since patients identified as frail by these indices tend to have poorer outcomes, it is crucial to recognize a change in their frailty. Hence it will be possible to adjust the treatment plan, to increase the intensity of therapy in those patients whose health status has improved and reducing it in those whose health has declined. Overall, there is a lack of data in MM, in both clinical trials and real-world analyses concerning changes in frailty status over time. In fact, most clinical studies assess frailty only once, either for inclusion or subgroup analysis, leaving the concept of frailty’s evolution over time largely unexplored in MM. To cover this clinical gap, Mian and colleagues utilized a comprehensive administrative database encompassing 4617 patients and employed a cumulative deficit model to categorize frailty dynamics. They investigated the association between frailty and survival outcomes and evaluated factors contributing to the worsening of frailty in a cohort of elderly patients with MM. Their findings revealed that the frailty status of more than 93% of patients either improved or worsened, noting that the most significant changes were observed within the first year of follow-up. As for therapy, it was observed that monotherapy with immunomodulatory drugs is helpful in protecting against additional declines in frailty. On the other hand, patients treated with a combination of an immunomodulatory agent and a proteasome inhibitor were at a higher risk of increasing frailty. This might be attributed to a more severe disease or to the major toxicity associated with the combination therapy, thus accelerating frailty. The current level of frailty is a more accurate predictor of outcomes than the initial frailty status as determined at diagnosis, underscoring the necessity for multiple assessments in this vulnerable group of patient and capable of reflecting changes over time [85].

### 2.2. Other Evidence from Clinical Trials

#### 2.2.1. The COACH Clinical Trial

The COACH clinical trial [86], a large-scale, multicenter cluster-randomized study, showed that GAM given to oncologists along with recommendations might help in improving the way they communicate issues related to aging. This approach enhances both patient and caregiver satisfaction with communication and care [87,88]. As outlined in the ASCO guidelines for geriatric oncology and supported by systematic reviews, deficiencies identified by the GA are linked to adverse events from chemotherapy, decreased completion rates of treatment, decline in function, earlier death, and higher consumption of healthcare resources. The COACH study recruited 541 elderly patients who were vulnerable and had either solid tumors or advanced lymphoma, all with at least one impaired GAM domain. Patients were randomly divided into two groups: one group received an “intervention” consisting of personalized GA summaries with tailored recommendations at oncology clinics, while the other group received “usual care”. The main goal was to evaluate if a summary of the geriatric assessment and recommendations given to hematologists would enhance discussions about issues related to aging. Secondary outcomes were the number of aging-related issues discussed during the visits, the quality of life, and caregivers’ satisfaction about communication on aging concerns. Study findings suggested that integrating a GAM into the clinical visits of elderly patients with advanced cancer resulted in a better communication, with a better focus on patient and caregiver’s concerns about aging [89].

#### 2.2.2. The GAIN Clinical Trial

The randomized trial GAIN [90] explored the interplay between severe toxicity chemotherapy-related and GAM in elderly patients with cancer. In this study, patients were randomly assigned in a standard of care (SOC) group and a GAIN group. The primary endpoint was the occurrence of grade ≥ 3 chemotherapy-related toxic effects, while secondary outcomes included emergency department admissions, average hospital stay, advance directive completion, unplanned hospitalizations, unplanned hospital readmissions, adjustments in chemotherapy doses, and early treatment discontinuations. In the GAIN group, the rate of grade ≥ 3 toxic effects were 50.5% as compared to 60.6% in the SOC group. GAIN also significantly increased the completion of advance directives to 28.4%, versus 13.3% in the SOC group, and no further differences were noted in the secondary outcomes between the groups. This study highlighted that the adoption of the multidisciplinary GAIN approach significantly lowered the occurrence of severe chemotherapy-related adverse events by more than 10%. Therefore, interventions driven by a geriatric assessment and carried out by a multidisciplinary team might reduce the harmful effects of chemotherapy without the need to decrease the scheduled dose. Survival rates at 6 and 12 months remained consistent across both groups, assuring that these GAM-driven interventions can reduce the adverse effects of chemotherapy without affecting its effectiveness [1].

## 3. Conclusions

Clinical evidence supporting GAM is almost evident for patients with solid tumors or lymphomas who are receiving chemotherapy treatment. However, there is also emerging evidence suggesting its utility in patients with other hematologic malignancies [90,91,92]. Using three streamlined geriatric assessment instruments (ADL, IADL, and CIRS-G) that were validated within an external cohort and linked to patient age, the Italian lymphoma group classified elderly patients with diffuse large B-cell lymphoma into the groups fit, unfit, and frail, and used this characterization to identify the correct treatment strategy [65,66,67,68,69].

A significant subset of older patients will now undergo immunotherapies and they could benefit from a geriatric assessment to better identify any management need. While the advantages of traditional chemotherapy are well-documented, much less exists about newer treatment strategies such as targeted therapies, immunotherapies, and bone marrow transplants. Hence, this new scenario warrants more detailed investigations. Even if many studies recognize the utility of GAM as a tool for assessing risk and averting negative outcomes, it would be interesting to establish how often the geriatric assessment should be assessed and at which time points patients should be re-assessed [93,94,95]. Moreover, many other vulnerable groups defined by gender, disability, country, non-English language usage, and other discriminative factors are also underrepresented in clinical studies [96,97]. These factors may often overlap with age, resulting in multiple layers of systemic exclusion. Future studies in older adults with hematologic cancers should not only assess the efficacy of various geriatric-focused intervention models but also examine their effects on treatment choices and patients’ outcomes. It will be helpful to identify which items of GAM-driven interventions are most beneficial. This knowledge will ameliorate clinical practices and the treatment of elderly patients with blood cancers [7,10,98]. A new promising frontier in cancer research and treatment especially for elderly patients is machine learning, which seems to offer potential revolutions in early diagnosis methodologies, individualizations of therapies, and monitoring disease progression [35,36]. Sophisticated algorithms can analyze large data sets (including clinical, genomic, proteomic, and imaging data) and may reveal patterns and correlations that would elude conventional human analysis. Therefore, in blood diseases, machine learning might be helpful in improving the accuracy and the interpretation of medical images [99,100], in analyzing genetic sequences to recognize mutations, and in contributing to the personalization of treatment care, especially in old people with comorbidities. By analyzing several patterns of patient data, algorithms can predict which patients will respond best to certain treatments, which may be at risk for serious side effects, or which might benefit from alternative treatment approaches. This customization will not only increase the effectiveness of treatment, but also minimize the likelihood of complications and improve the overall patient experience [101,102]. Ultimately, this technology might support oncology research by providing new insights into the molecular and cellular mechanisms of cancer, accelerating the development of new drugs and therapies [103,104]. The growing adoption of machine learning in elderly patients with hematological malignancies promises to improve survival rates and quality of life, as well as making treatments more efficient and accessible everywhere.

## Figures and Tables

**Table 1:** Domains included in a geriatric assessment management used in patients with cancer.

Components of a Geriatric Assessment Management	
Domain	Example
Function and mobility	Assess difficulty in activities such eating and dressing, assess gait speed and strength
Comorbidities	Assess the presence of other diseases
Social functioning and support	Assess the presence of social support in daily activities
Cognition	Assess memory, concentration, and orientation
Psychological problems	Assess anxiety and depression
Nutrition	Assess actual weight and weight changes in the last six months
Polypharmacy	Assess high-risk medications and regularly scheduled medications

**Table 2:** Examples of geriatric assessment tools used in hematological malignancies.

	Domains	Purpose	Hematological Malignancies
**Geriatric 8 Screening Tool**	Weight loss, age, psychological status, number of medications, body mass index	Predict overall-survival and treatment toxicity	Non-Hodgkin Lymphoma, Myelodysplastic Syndromes, Leukemia
**Geriatric Assessment in Hematology**	Self-rated health status, nutrition, comorbidities, number of medications, psychological status	Identify frail patients	Multiple Myeloma, Myelodysplastic Syndromes, Leukemia
**Elderly Prognostic Index**	ADL, age, hemoglobin level, comorbidities, IADL	Identify frail patients with DLBCL and predict their overall survival	DLBCL
**Vulnerable Elders Survey-13**	Functional limitations, functional disabilities, age	Evaluate the risk of functional status deterioration and predict overall-survival	Non-Hodgkin Lymphoma

## Abbreviations

aaIPI, age-adjusted international prognostic index; ACE-27, Adult Comorbidity Evaluation-27; ADLs, activities of daily living; ASCO, American Society of Clinical Oncology; BOMC, Blessed Orientation Memory Concentration; CCI, Charlson Comorbidity Index; CGA, Comprehensive geriatric assessments; CIRS, Cumulative Illness Rating Scale; CIRS-G, Cumulative Illness Rating Scale for Geriatrics; CML, chronic myeloid leukemia; CRASH, Chemotherapy Risk Assessment Scale for High-Age Patients; CTCAE, Common Toxicity Criteria for Adverse Events; DAS, dasatinib; DLBCL, diffuse large B-cell lymphoma; DT, Distress Thermometer; EFS, event-free survival; ELFI, Elderly Functional Index; FU, follow-up; G8, Geriatric 8; GA, Geriatric Assessment; GAM, geriatric assessment management; GDS, Geriatric Depression Scale; HCT-CI, Hematopoietic Cell Transplantation-Comorbidity index; HRQOL, health-related quality of life; iADLs, instrumental activities of daily living; ICU, intensive care unit; IM, imatinib; IMWG, International Myeloma Working Group; INTEGERATE, Integrated Geriatric Assessment and Treatment Effectiveness; LMICs, low- and middle-income countries; MAI, Medication Appropriateness Index; ML, machine learning; MM, multiple myeloma; MMSE, Mini-Mental State Examination; MNA, Mini-Nutritional Assessment; MoCA, Montreal Cognitive Assessment; MOS, Medical Outcomes Study; RDDI, relative doxorubicin dose intensity; SOC, standard of care; SPPB, Short Physical Performance Battery; START, Screening Tool to Alert Doctors to Right Treatment; STOPP, Screening Tool of Older Persons’ Potentially Inappropriate Prescriptions; TFR, treatment-free remission; TKIs, tyrosine kinase inhibitors; TUG, Timed Up and Go; VES-13, Vulnerable Elders Survey-13.
